# Premature atrial contractions with multiple patterns of aberrant conduction followed by torsade de pointes in a patient with polymyalgia rheumatica: A case report

**DOI:** 10.1097/MD.0000000000027286

**Published:** 2021-09-17

**Authors:** Koji Takahashi, Mina Yamashita, Tomoki Sakaue, Daijiro Enomoto, Shigeki Uemura, Takafumi Okura, Shuntaro Ikeda, Masafumi Takemoto, Yutaka Utsunomiya, Takashi Hyodo, Masayuki Ochi, Satoshi Higuchi

**Affiliations:** a Department of Community Emergency Medicine, Ehime University Graduate School of Medicine, Ehime, Japan; b Department of Cardiology, Yawatahama City General Hospital, Ehime, Japan; c Department of Medical Engineering, Yawatahama City General Hospital, Ehime, Japan; d Department of Geriatric Medicine and Neurology, Ehime University Graduate School of Medicine, Ehime, Japan; e Higuchi Internal Medicine Clinic, Ehime, Japan.

**Keywords:** β-blocker, electrical storm, interleukin-6, polymyalgia rheumatica, premature atrial contraction, torsade de pointes

## Abstract

**Rationale:**

Recent studies have shown that QT interval prolongation is associated with disease severity and predicts mortality in systemic inflammatory diseases, particularly rheumatoid arthritis. Systemic pro-inflammatory cytokines released from synovial tissues in rheumatoid arthritis, such as interleukin (IL)-1β, IL-6, and tumor necrosis factor-α, could have direct effects on cardiac electrophysiology, particularly changes in the expression and function of potassium and calcium channels, resulting in QT interval prolongation on surface electrocardiogram (ECG) and an increased predisposition to develop lethal ventricular arrhythmias. However, reports on torsade de pointes (TdP) due to acquired long QT syndrome in patients with polymyalgia rheumatica (PMR) are limited.

**Patient concerns:**

An 85-year-old Japanese woman with active PMR developed first syncope.

**Diagnosis:**

Frequent premature atrial contractions (PACs) with multiple patterns of aberrant conduction, QT interval prolongation, and morphological T-U wave variability followed by TdP were documented. PACs were the first beat of TdP.

**Interventions:**

Amiodarone, together with magnesium and potassium, was intravenously administered. However, TdP resulted in a ventricular arrhythmic storm, for which sedation with mechanical ventilatory support, temporary overdrive cardiac pacing, and intravenous landiolol administration in addition to multiple direct current shocks were effective.

**Outcomes:**

Approximately 2 years later, the patient was treated with amiodarone, propranolol, and prednisolone. She did not undergo implantable cardioverter-defibrillator implantation and was quite well, with no recurrence of ventricular tachyarrhythmia.

**Lessons:**

IL-6 hyperproduction in inflamed tissues has been widely confirmed in PMR. Frequent PACs with various patterns of aberrant conduction, QT interval prolongation, and morphological T-U wave variability followed by TdP, for which IL-6-mediated enhancement of L-type Ca^2+^ current and inhibition of the rapid component of the delayed rectifier K^+^ current are the most likely mechanisms, were documented in an elderly Japanese woman with PMR. ECG may be recorded once in patients with active PMR even when these patients do not complain of palpitation or syncope. If QT interval prolongation or arrhythmia, including even PACs, is observed, follow-up ECG may be warranted, particularly for patients with some risk factors for QT prolongation that could lead to TdP, such as advanced age, female sex, hypopotassemia, and polypharmacy.

## 1 Introduction

Premature atrial contractions (PACs) are common in all ages and increase with age.^[1]^ PACs are commonly idiopathic in the setting of a structurally normal heart, and the structural causes of PACs include various heart diseases. Medical pathologies associated with increased PACs include hypertension, diabetes mellitus, chronic obstructive pulmonary disorder, infection, and inflammation.^[1,2]^ Many PACs are considered benign electrophysiological phenomena, and prognosis is generally dependent on the underlying cause of the PACs and the presence of structural cardiac disease, although PACs originating in the pulmonary veins are initiators of most atrial fibrillations and have been shown to be associated with a greater risk of ischemic stroke development.^[3]^

Recent studies have shown that the QT interval prolongation is associated with disease severity and inflammatory markers, and predicts mortality in systemic inflammatory diseases, particularly rheumatoid arthritis.^[4]^ Systemic pro-inflammatory cytokines released from synovial tissues in rheumatoid arthritis, such as interleukin (IL)-1β, IL-6, and tumor necrosis factor-α, could have direct effects on cardiac electrophysiology, particularly changes in the expression and function of potassium and calcium channels, resulting in a prolonged effect on the cardiomyocyte action potential duration and thereby affecting the QT interval prolongation on surface electrocardiogram (ECG) and an increased predisposition to develop lethal ventricular arrhythmias. However, reports on torsade de pointes (TdP) due to acquired long QT syndrome (LQTS) in patients with polymyalgia rheumatica (PMR), which is a common, chronic, systemic rheumatic inflammatory disease that affects adults older than 50 years,^[5]^ are limited.^[6]^

We encountered a case of active PMR in which frequent PACs with multiple patterns of aberrant conduction (such as right bundle branch block [RBBB], left anterior fascicular block [LAFB], left septal fascicular block [LSFB], or left posterior fascicular block [LPFB]), QT interval prolongation, and morphological T-U wave variability followed by TdP polymorphic ventricular tachycardia that led to an electrical storm were documented.^[7–9]^ PACs were the first beat of TdP. Informed consent was obtained from the patient for the publication of this case report and accompanying images.

## 2 Case presentation

**Figure 1:**
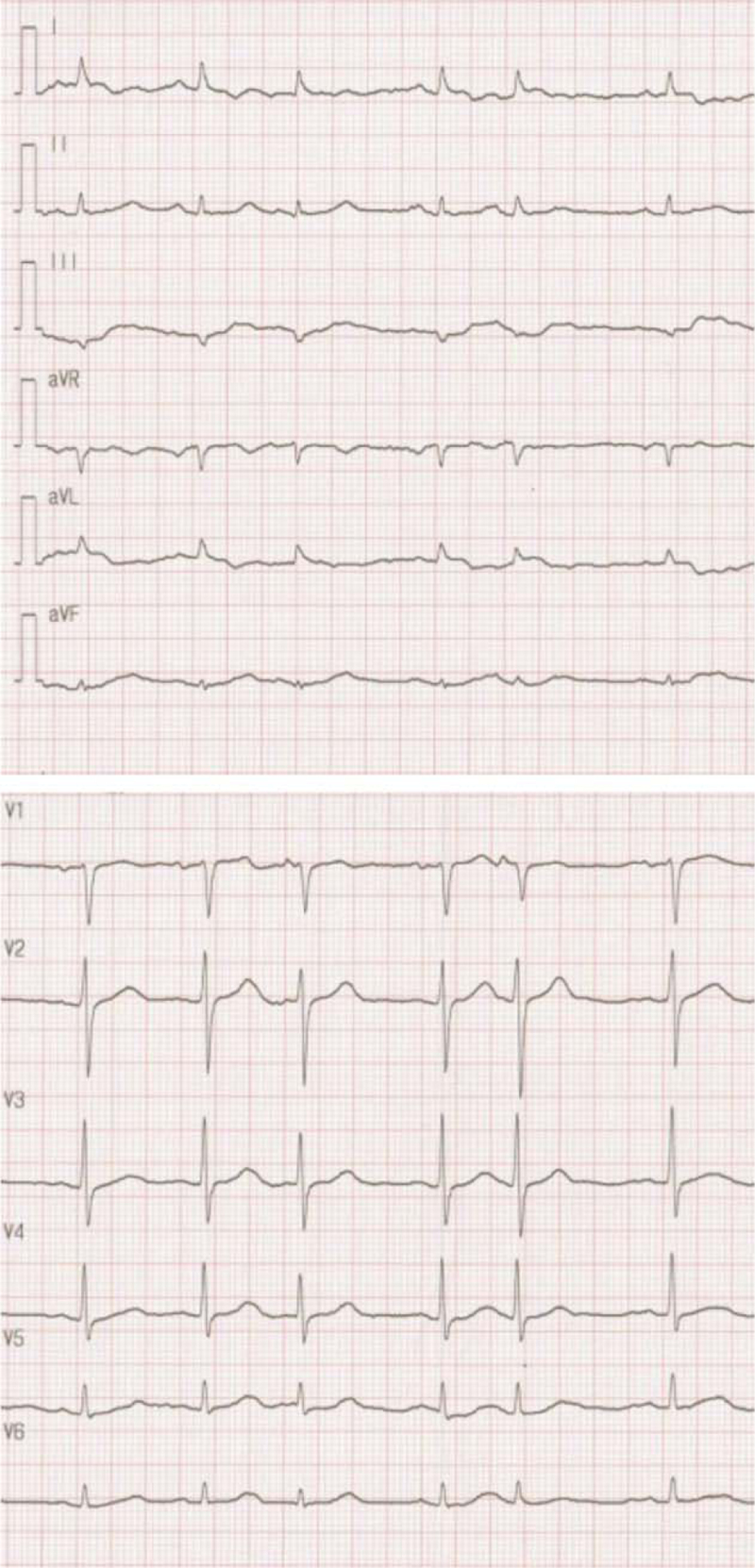
An electrocardiogram (ECG) recorded in another clinic at 1 yr before admission. The ECG shows sinus rhythm and multiple premature atrial contractions (PACs) with no aberrant conduction. The coupling interval between the PAC and the preceding sinus beat is not fixed. The heart rate-corrected QT intervals, calculated using the Bazett formula to adjust the measured QT interval for cycle length by dividing the observed uncorrected QT interval by the square root of the R-R interval, are 470 ms.

An 85-year-old Japanese woman was transferred by ambulance to Yawatahama City General Hospital because of transient loss of consciousness. She had no history of smoking or alcohol consumption. Her medical history included long-standing hypertension, dyslipidemia, bronchial asthma, chronic renal disorder, hyperuricemia, and silent cerebral infarction, but she had no angina pectoris. She had been prescribed oral valsartan (40 mg/day), amlodipine besylate (7.5 mg/day), trichlormethiazide (1 mg/day), atorvastatin calcium hydrate (5 mg/day), and cilostazol (200 mg/day) at another clinic. Dry powder inhalers containing budesonide and formoterol fumarate dihydrate (2 puffs/day) were also prescribed. She underwent bilateral total knee arthroplasty for osteoarthritis at 10 years prior. Her ECG recorded in the clinic at 1 year prior revealed slight QT interval prolongation and multiple PACs without aberrant conduction (Fig. 1).

**Figure 2:**
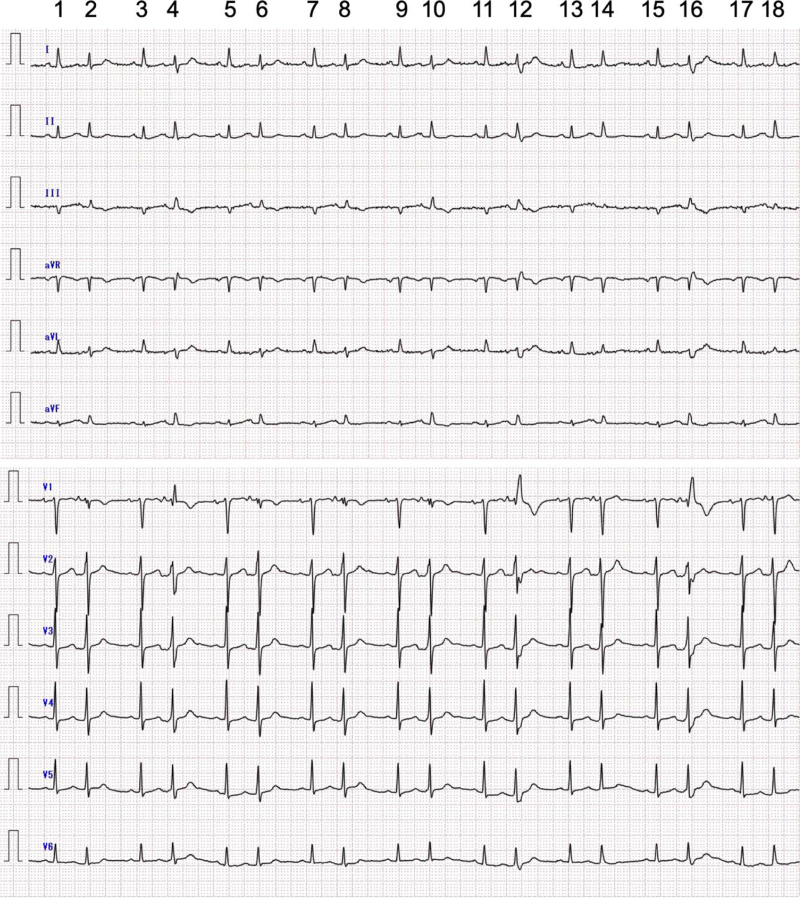
An electrocardiogram (ECG) recorded at 7 d before admission. The ECG shows sinus rhythm and atrial bigeminy. The coupling interval, defined as the interval between a premature atrial contraction (PAC) and a preceding sinus beat, is almost fixed at 450 ms. The P-wave duration is 90 ms in sinus beats and 100 ms in PACs. The PR duration is 140 ms for sinus beats and 110 ms for PACs. Some PACs have aberrant conduction of incomplete (the 4th beat) or complete right bundle branch block (RBBB) (the 12th and 16th beats) and left posterior fascicular block morphology. The QRS-ST-T morphology in the remaining PACs is also slightly different from that in sinus beats, indicating mild aberrant conduction; a slight shift of the QRS axis in the frontal plane and rsr's’ pattern in lead V1 together with an increase in the depth of s-waves in the left precordial leads are seen in the 2nd, 6th, 8th, and 10th beats, though to reflect incomplete RBBB-like morphology with disappearance of “incomplete” left anterior fascicular block. In PACs, the QT interval and the T_peak_–T_end_ interval are approximately 370 and 110 ms, respectively, but T-waves merge with U-waves in some PACs (the 12th, 14th, and 16th beats).

At 7 days prior, she visited the Department of Neurology and the Department of Surgery at Yawatahama City General Hospital because of headache and stiffness in the neck and bilateral shoulders lasting for several weeks. At that time, her temperature was 37.6°C; pulse rate, 57 beats/minute with bigeminal rhythm; systemic blood pressure, 150/55 mm Hg; and oxygen saturation on room air measured using a pulse oximeter, 96%. Her blood tests revealed marked inflammatory response (C-reactive protein, 20.84 mg/dL; erythrocyte sedimentation rate, 99 mm/hour), microcytic hypochromic anemia with hemoglobin concentration of 9.8 g/dL, and renal dysfunction with an estimated glomerular filtration rate of 15 mL/minute/1.73 m^2^. The serum potassium concentration was 4.4 mEq/L. ECG revealed sinus rhythm and atrial bigeminy. Some PACs had incomplete or complete RBBB and RBBB-like QRS configuration, which may be due to aberrant conduction (Fig. 2). The echocardiogram showed a normal left ventricular ejection fraction of 64%, a slightly dilated left ventricle with an end-diastolic dimension of 48.3 mm, a slightly dilated left atrium with a volume of 58.1 mL/m^2^, and a normal interventricular septal wall thickness of 7.8 mm. Moderate aortic valvular stenosis with a maximum aortic jet velocity of 3.6 m/second, mean transvalvular pressure gradient of 25 mm Hg, or continuity equation valve area of 1.0 cm^2^ assessed by Doppler echocardiography was observed. Oral acetaminophen (1500 mg/day) was administered. Thereafter, the patient was diagnosed with PMR, and acetaminophen was replaced with prednisolone (20 mg/day).

In the emergency room, her temperature was 36.5°C, pulse rate was 51 beats/minute, systemic blood pressure was 161/52 mm Hg, respiratory rate was 18 breaths/minute, and oxygen saturation was 100% at a flow rate of 3 L/minute over an oxygen mask. On auscultation, there was a systolic ejection murmur of grade 4/6, but with no rales in the lung fields. The liver was palpable with a two-finger breadth along the right midclavicular line below the costal margin. Mild pretibial edema was observed.

**Figure 3:**
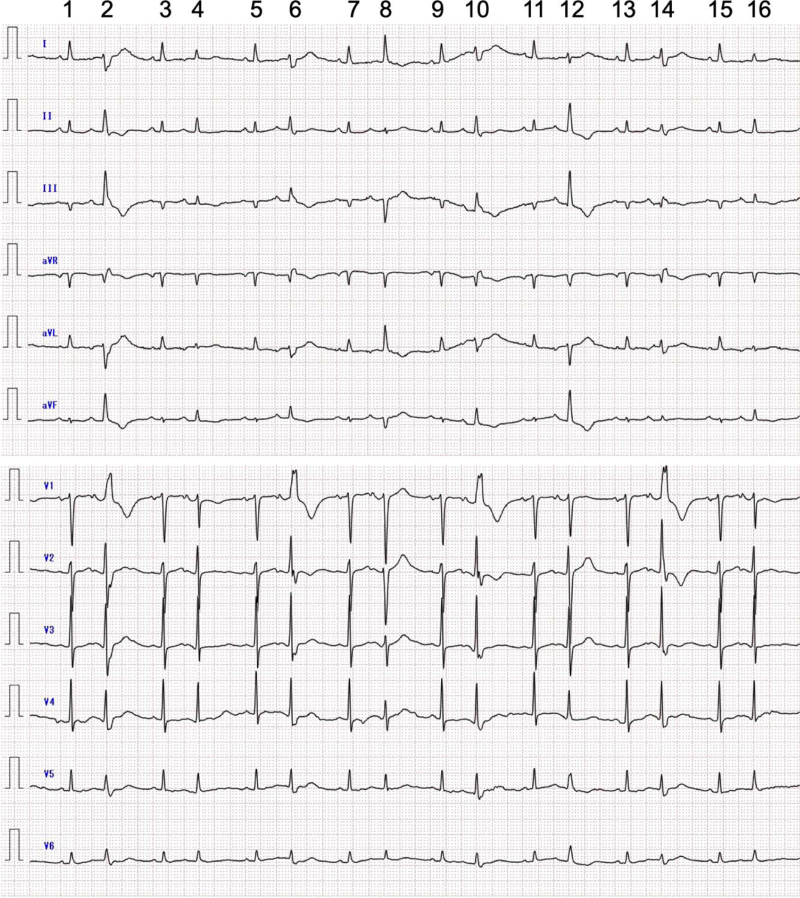
Electrocardiogram (ECG) recorded at admission. The ECG shows sinus rhythm and atrial bigeminy. The coupling interval between a premature atrial contraction (PAC) and a preceding sinus beat is almost fixed at 460 ms. The P-wave duration is 95 ms in sinus beats and 90 ms in PACs. The PR duration is 145 ms in sinus beats and 160 ms in PACs, together with PR-segment depression in PACs. All PACs have aberrant conduction. In the 2nd, 6th, 10th, and 14th beats, complete right bundle branch block plus left posterior fascicular block (LPFB) morphology is seen together with the increased height of the R-waves in lead V2 compared to that in sinus beats suggestive of “incomplete” or “complete” left septal fascicular block (LSFB). In the 8th and 12th beats, left anterior fascicular block (LAFB) and LPFB morphologies are observed, respectively. A slight shift of the QRS axis in the frontal plane and the increased height of the R-waves in lead V2 compared to that in sinus beats, thought to reflect the disappearance of “incomplete” LAFB and appearance of “incomplete” LSFB, are seen in the 4th and 16th beats. In PACs, morphological T-U wave variability is observed, and QT (QU) intervals are fluctuated. The longest QT (QU) interval and the T_peak_–T_end_ interval are over 500 and 200 ms, respectively, although the end of the T-waves is difficult to determine because T-waves merge with U-waves in some PACs.

The blood tests revealed a high-sensitivity cardiac troponin I level of 12.7 pg/mL, which was below the reference range (≤16.0 pg/mL), but a brain natriuretic peptide level of 293.1 pg/mL, over the reference range (≤18.4 pg/mL). Her C-reactive protein level decreased to 3.02 mg/dL. The serum potassium concentration was 4.1 mEq/L, and magnesium concentration was not measured. A chest radiograph obtained with the patient in the supine position did not show pulmonary congestion. The ECG revealed sinus rhythm and atrial bigeminy with alternating RBBB morphology and non-RBBB morphology (Fig. 3). The echocardiogram showed no remarkable changes in findings that were recorded at 7 days prior.

**Figure 4:**
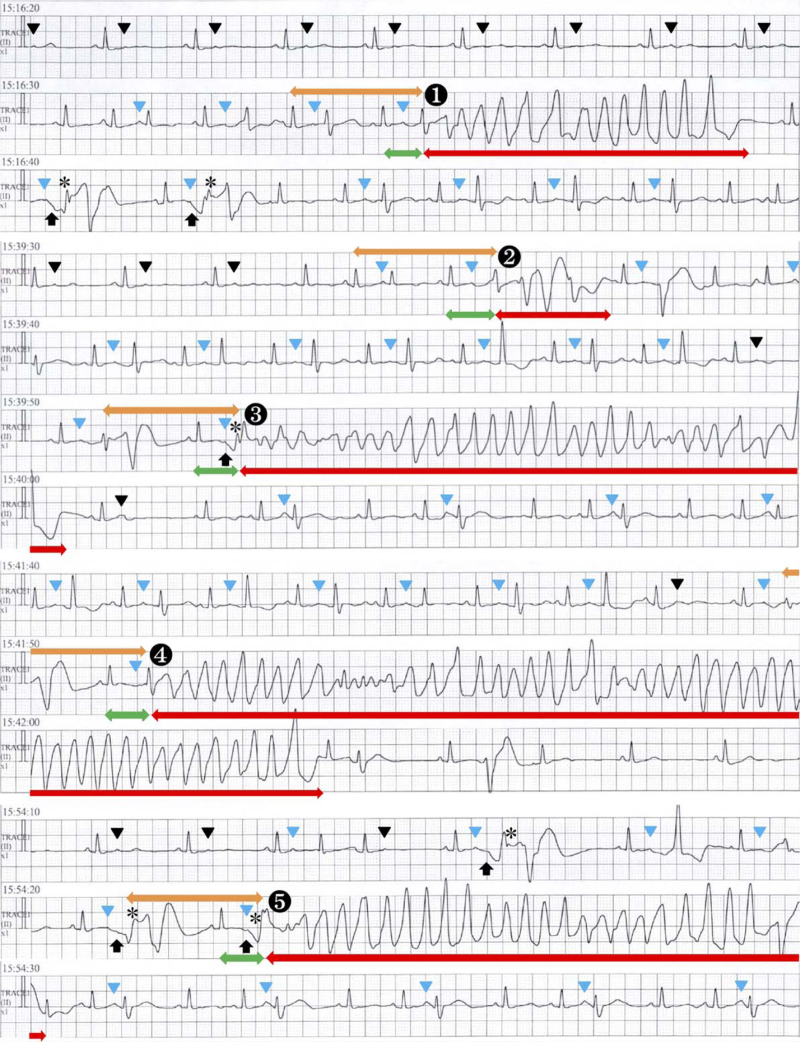
Rhythm strips of continuous electrocardiograms from bedside cardiac monitors recorded at approximately 1 h after admission. A bigeminal rhythm due to sinus beats and premature atrial contractions (PACs), including non-conducted PACs (black arrowheads), is observed. The QRS-ST configuration of PACs, arising from the nadir of giant T-U waves (black arrows), followed by a bizarre premature ventricular beat falling atop the T-wave, shows a massive ST-segment elevation thought to be caused by a transmural conduction block (asterisks). Additionally, five sets of torsade de pointes (TdP) non-sustained ventricular tachycardias are observed. There are two initiation patterns of TdP events with or without the QRS complex of the first TdP beat arising from giant negative T-U waves (black arrows). All TdP events are initiated with a short–long–short R–R cycle sequence and spontaneously terminated. PACs are the first beat of TdP. The coupling interval, defined as the interval between the first beat of TdP and the preceding sinus beat (double-headed green arrows), is over 500 ms, suggesting “true” TdP. The double-headed orange arrows and double-headed red arrows indicate the short–long–short R–R cycle sequence and TdP, respectively. Blue arrowheads indicate the conducted PACs. During sinus rhythm with non-conducted PACs in a pattern of atrial bigeminy, the QT, and heart rate-corrected QT (QTc) intervals of sinus beats are 600 and 550 ms, respectively. The QTc was calculated using the Bazett formula to adjust the measured QT interval for cycle length by dividing the observed uncorrected QT interval by the square root of the R–R interval.

Non-sustained TdP ventricular tachycardia was documented in the emergency room; thus, intravenous administration of amiodarone hydrochloride, together with magnesium and potassium, was started at a dose of 50 mg/hour without initial loading infusions of 125 mg over the first 10 minutes. Approximately 6 hours after starting intravenous administration of amiodarone hydrochloride, non-sustained TdP continued (Fig. 4). Thus, intravenous administration of amiodarone hydrochloride was stopped, and nifekalant hydrochloride was intravenously administered at 20 mg over the first 3 minutes, followed by 500 mg over the next 24 hours. However, multiple sustained ventricular tachycardias or ventricular flutter with hemodynamic collapse requiring direct current shocks to resuscitate developed. The patient was diagnosed with a ventricular electrical storm. First, deep sedation to alleviate the sympathetic overdrive by achieving Richmond Agitation and Sedation Scale values below −2 with the use of one-shot intravenous administration of thiopental sodium followed by continuous intravenous administration of dexmedetomidine hydrochloride, along with mechanical ventilation after orotracheal intubation, was performed. Second, temporary overdrive cardiac pacing was performed. Subsequently, the ventricular tachyarrhythmia disappeared. However, non-sustained ventricular tachycardia was documented at the time of stopping temporary pacing on the 2nd hospital day. Intravenous administration of nifekalant hydrochloride was discontinued, and intravenous administration of amiodarone hydrochloride was resumed at a maintenance dose of 600 mg/day. Moreover, intravenous administration of landiolol hydrochloride was started at 1.5 μg/kg/minute and increased by 1.5 μg/kg/minute every 30 minutes to a maximum of 10 μg/kg/minute without recurrence of ventricular tachyarrhythmia even after stopping cardiac pacing.

On the 6th hospital day, 30 mg/day propranolol hydrochloride was administered via a nasogastric tube, and the dose of landiolol hydrochloride was initially reduced from 10 μg/kg/minute to 7 μg/kg/minute, then from 7 μg/kg/minute to 5 μg/kg/minute, and subsequently by 1 μg/kg/minute increments every 24 hours. The patient ultimately stopped landiolol hydrochloride treatment without recurrent ventricular extrasystoles. On the 8th hospital day, amiodarone hydrochloride was continued with a dose reduction from 600 mg/day administered intravenously to 200 mg/day administered via a nasogastric tube. On the 9th hospital day, the patient was weaned off the mechanical ventilator and extubated after stopping intravenous administration of dexmedetomidine hydrochloride.

**Figure 5:**
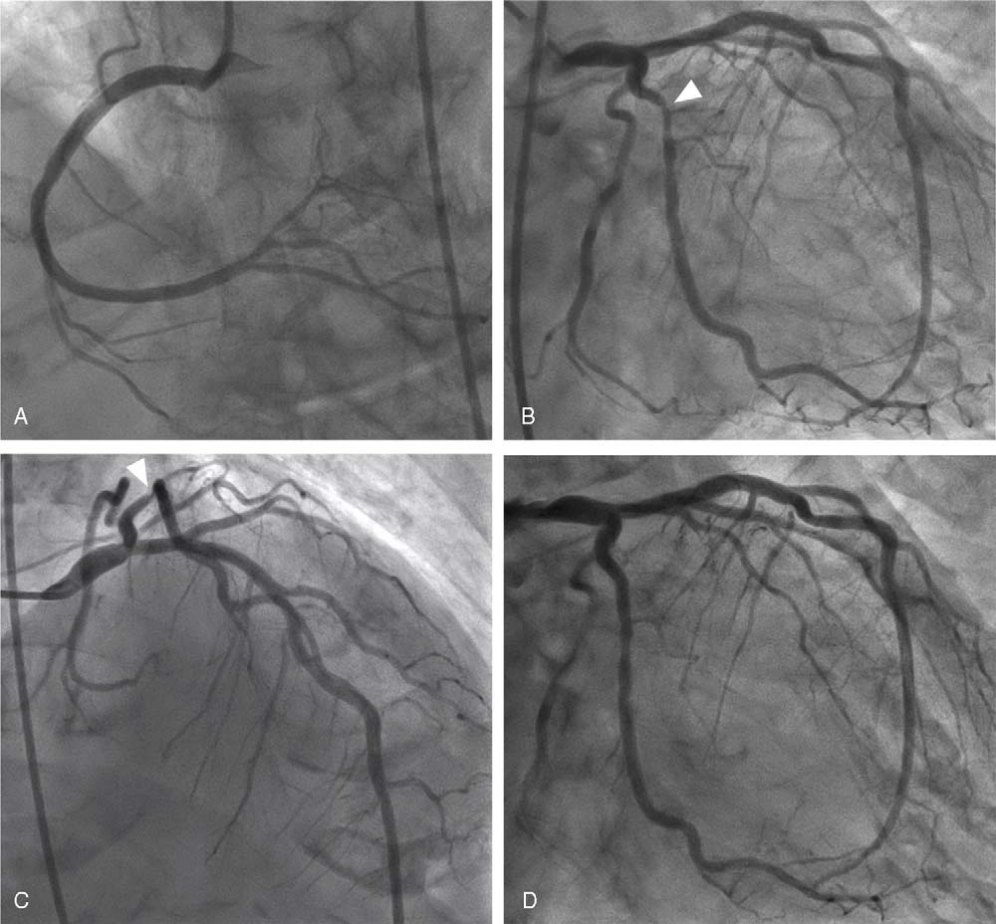
Coronary angiograms. Coronary angiograms reveal severe narrowing without thrombi or washout delay of the contrast medium at the posterolateral branch of the left circumflex coronary artery (LC) (panels B and C), but no significant stenosis is found in the right coronary artery or left anterior descending coronary artery (panels A–C). A coronary stent is successfully deployed to the lesion without residual stenosis (panel D). Arrowheads indicate severe stenosis of the LC.

Cardiac catheterization was performed on the 17th day of hospitalization. She had a cardiac index of 3.90 L/minute/m^2^ and a mean pulmonary capillary wedge pressure of 13 mm Hg. Coronary angiography revealed a narrowing without thrombi in the proximal left circumflex coronary artery (Fig. 5). The instantaneous wave-free ratio obtained with the use of a coronary-pressure guidewire was 0.84; thus, the patient was diagnosed with silent myocardial ischemia. An intravascular ultrasonography-guided everolimus-eluting platinum chromium coronary stent was successfully deployed to the lesion without residual stenosis. Diagnostic genetic testing for LQTS revealed a p.Ala283Val missense variant in KCNQ1.

Approximately 2 years later, the patient was treated with amiodarone (50 mg/day), propranolol (30 mg/day), and prednisolone (10 mg/day). She did not undergo implantable cardioverter-defibrillator implantation and was quite well, with no recurrence of ventricular tachyarrhythmia.

## 3 Discussion

The points of this case report are as follows:

(i) frequent PACs with various patterns of aberrant conduction, QT interval prolongation, and morphological T-U wave variability were observed;
(ii) TdP was induced by PAC but not premature ventricular contraction; and
(iii) TdP occurrence could be associated with the disease activity of PMR.

The most noticeable ECG finding in our patient was frequent PACs with various QRS-ST-T configurations thought to be RBBB, LAFB, LSFB, LPFB, or a combination of these blocks (Fig. 3).^[7,8]^ In general, these morphological changes in PACs may be due to aberrant ventricular conduction. If a PAC occurs while the atrioventricular node and His-Purkinje system are still refractory, the conducted beat will become aberrant. As the refractory period of the right bundle is slightly longer than that of the left bundle, the aberrantly conducted beat typically shows an RBBB morphology. In 1976, Kulbertus et al reported that multiple patterns of aberrant ventricular conduction were induced in several patients after the introduction of PACs through a transvenous electrode catheter.^[10]^ In our patient, PACs with multiple patterns of aberrant conduction, including LAFB alone, RBBB plus LPFB, RBBB plus LPFB and LSFB, were spontaneously documented and were not by an experiment. Moreover, Kulbertus et al also pointed out that some patterns of aberrant conduction could not be readily classified into the usual categories of intraventricular conduction defects,^[10–12]^ despite being obviously different in shape from the control (Figs. 2 and 3). The degree of aberrancy seemed to worsen for only a week (Figs. 2 and 3). The coupling intervals between PACs and preceding sinus beats were fixed even on admission, indicating unifocal PACs, and were almost the same as those at 7 days prior. Thus, these morphological changes in QRS-ST-T configurations in PACs could be unlikely due to Ashman's phenomenon as a physiological aberration and have a pathologic nature because the conduction disturbance does not comply with the “aberrancy rule.” While PACs as an initiation of ventricular tachyarrhythmia are uncommon, the atrial impulse propagates through the atrioventricular node and into the cardiac ventricles; such atrial impulse can induce ventricular tachyarrhythmia.^[13,14]^

In our patient, frequent PACs were documented at 1 year prior to TdP occurrence. Thus, we failed to establish any of the abovementioned triggering factors for PACs.^[1,2]^ However, frequent PACs with QT interval prolongation, prolonged T_peak_–T_end_ interval, and morphological T-U wave variability reflecting the spatial dispersion of ventricular repolarization used as non-invasive markers for predicting the risk of malignant cardiac arrhythmias were thought to result from active inflammation due to PMR (Fig. 3).^[9,15]^ Nonetheless, accurate QT interval assessment appeared to be difficult because almost all heartbeats showed sinus rhythm with PACs in a pattern of atrial bigeminy. In general, extra systoles and the beat directly following an extra systole should not be included in QT interval measurement.^[16]^ In PMR, the role of circulating IL-6, mainly produced in inflamed tissues, appears dominant, and IL-6 hyperproduction has been widely confirmed. Circulating IL-6 prolongs the action potential duration by enhancing L-type Ca^2+^ current (I_Ca,L_), which causes phase 2 early afterdepolarization.^[17]^ Moreover, IL-6 inhibits the rapid component of the delayed rectifier K^+^ current (I_kr_), prolonging the action potential duration and QT interval.^[18]^ Although serum IL-6 levels were not measured, IL-6-mediated enhancement of I_Ca,L_ and inhibition of I_Kr_ are the most likely mechanisms for QT interval prolongation and morphological T-U wave variability in our patient, leading to TdP. Moreover, most patients with acquired LQTS have one or more risk factors, such as advanced age, female sex, hypopotassemia, latent congenital LQTS, and polypharmacy.^[19]^ In our elderly female patient, the QT interval was slightly prolonged at 1 year prior (Fig. 1), and diagnostic genetic testing for LQTS revealed the p.Ala283Val missense variant in KCNQ1. Nevertheless, we consider that this is a variant of uncertain significance because there is insufficient information to support a more definitive classification of this variant at this time. On the contrary, cilostazol, which was prescribed at another clinic, is a selective phosphodiesterase type III inhibitor that has a likely mechanism for QT interval prolongation resulting from I_Ca,L_-mediated action potential prolongation.^[20]^

In our patient, all TdP events had a short–long–short sequence preceding TdP. In these TdP events, the main mechanism underlying QT interval prolongation was likely I_Kr_ blockade.^[21]^ Furthermore, the coupling interval, defined as the interval between the first beat of TdP and the preceding sinus beat, was over 500 millisecond, suggesting “true” TdP related to QT interval prolongation^[22]^; however, there were two initiation patterns of TdP events (Fig. 4). In the third and fifth TdP events, the QRS complex of the first TdP beat arose from giant negative T-U waves^[19,23]^; conversely, in the first, second, and fourth TdP events, it did not. The latter might be a short-coupled variant of TdP with a coupling interval of approximately 300 millisecond to the preceding PAC, considering the difficulty in determination by ECG monitoring only. If so, these TdP events signify Purkinje-related ventricular tachyarrhythmias.^[24]^ Purkinje cells differ from ventricular myocytes with respect to electrical membrane properties, ionic currents, and ion-channel expression, with the former being more arrhythmogenic than the latter.^[25]^ The most notable difference lies in Ca^2+^ handling; distinct proarrhythmic Ca^2+^-mediated mechanisms can lead to the ectopic afterdepolarization of Purkinje cells related to a highly prevalent triggered activity. In our patient, the PR duration in PACs on admission was longer than that at 7 days prior, irrespective of the absence of an interval change in P-wave duration (Figs. 2 and 3). This indicates a conduction abnormality in the atrioventricular node and His-Purkinje network, resulting in a preferential anterograde block, allowing retrograde conduction only.^[25]^ Aberrant excitation patterns can also lead to a unidirectional block, which predisposes to re-entry formation. IL-6-mediated enhancement of I_Ca,L_ and inhibition of I_Kr_ in PMR, as well as that of ventricular myocytes, also prolong the action potential duration of Purkinje cells. Even in LQTS, Purkinje tissues are thought to be an important trigger of TdP.^[26]^

A ventricular electrical storm, which is a state of cardiac electrical instability, is a life-threatening syndrome characterized by clustering of recurrent episodes of ventricular tachyarrhythmia within a relatively short period of time.^[27,28]^ Acute management is aimed at reducing the burden of ventricular tachyarrhythmias and improving survival.^[29]^ Triggers for electrical storms, such as myocardial ischemia as shown in our patient, acute heart failure, electrolyte disorders, hypoxia, and drug-related arrhythmogenicity, have to be screened for and immediately corrected,^[30]^ although a trigger is identified only in 10% to 15% of patients.^[28]^ An anti-arrhythmic drug regimen of β-blockers, amiodarone, sotalol, lidocaine, or quinidine should be considered in the acute phase to suppress further ventricular tachyarrhythmias. These drugs may be sequentially administered or a selection of them may be combined, if needed, with caution owing to the risk of potential proarrhythmia. In our patient, sustained ventricular tachyarrhythmias developed after intravenous administration of nifekalant hydrochloride (an I_Kr_-selective blocking agent), which was switched from amiodarone hydrochloride, and might be due to the proarrhythmic reaction of these drugs.^[31]^ Hendriks and Szili-Torok recommend that the first step in the treatment of electrical storm is the administration of β-blockers, which play a fundamental role in the management of electrical storms by blocking the sympathetic system.^[29]^ Furthermore, they mentioned that propranolol, a lipophilic unselective β-blocker, was more effective in suppressing ventricular tachyarrhythmias than metoprolol and amiodarone. In our patient, landiolol hydrochloride, an ultra-short-acting β1-superselective β-adrenergic blocker, was considered highly effective, although we prescribed propranolol hydrochloride in the subacute to chronic phase. If we administered a β-adrenergic blocker to the patient before sustained ventricular tachycardia developed, an electrical storm might not occur. Moreover, calcium channel blockers, such as verapamil, might be effective in suppressing I_Ca,L_ via IL-6 hyperproduction in patients with PMR. Verapamil has been reported to be effective against some types of acquired LQTS.^[32]^ When a ventricular electrical storm remains intractable despite aggressive anti-arrhythmic therapies, deep sedation must be considered, along with mechanical ventilation.^[33]^ High-rate pacing for pause-dependent TdP, such as LQTS-related TdP as shown in our patient, would be effective, and administration of β-blockers or calcium channel blockers under temporary pacing would be preferable to prevent pauses that trigger TdP.^[19,21]^ We considered that ventricular tachyarrhythmias could be suppressed by drug therapy; hence, implantation of an implantable cardioverter-defibrillator as the first-line treatment option for secondary prevention against sudden cardiac death was not required in our patient.

In conclusion, frequent PACs with various patterns of aberrant conduction, QT interval prolongation, and morphological T-U wave variability, for which IL-6-mediated enhancement of I_Ca,L_ and inhibition of I_Kr_ are the most likely mechanisms, were the first beats of TdP in an elderly Japanese woman with PMR. Irrespective of an intravenous administration of anti-arrhythmic drugs together with magnesium and potassium, TdP resulted in a ventricular arrhythmic storm. PACs in our patient might be “malignant” ectopies, which masquerade as “benign” beats, leading to lethal ventricular tachyarrhythmia. ECG may be recorded once in patients with active PMR even when these patients do not complain of palpitation or syncope. If QT interval prolongation or arrhythmia, including even PACs, is observed, follow-up ECG may be warranted, particularly for patients with some risk factors for QT prolongation that could lead to TdP, such as advanced age, female sex, hypopotassemia, latent congenital LQTS, underlying structural heart diseases, impairment in hepatic drug metabolism, and polypharmacy. Further studies involving larger case numbers are necessary to clarify the relationship between the QT interval and disease activity in patients with PMR.

## Acknowledgments

The authors thank Editage (www.editage.com) for English language editing.

## Author contributions

**Conceptualization:** Koji Takahashi.

**Data curation:** Koji Takahashi, Mina Yamashita, Tomoki Sakaue, Daijiro Enomoto, Masayuki Ochi.

**Investigation:** Koji Takahashi.

**Methodology:** Koji Takahashi.

**Resources:** Masafumi Takemoto, Yutaka Utsunomiya, Takashi Hyodo, Masayuki Ochi, Satoshi Higuchi.

**Supervision:** Koji Takahashi, Shigeki Uemura, Takafumi Okura, Shuntaro Ikeda.

**Visualization:** Koji Takahashi.

**Writing – original draft:** Koji Takahashi, Takafumi Okura, Shuntaro Ikeda.

**Writing – review & editing:** Koji Takahashi, Takafumi Okura, Shuntaro Ikeda.
